# Corpora Lutea

**Published:** 1843-01

**Authors:** 


*Corpora Lutea.*—Dr. Wm. Davidson of Edinburgh, gives an account of three dissections of females, neither of whom was pregnant, and in each of which, corpora lutea were found. They had all the characters assigned to them by Dr. Montgomery; a central cavity or fibrous coagulum; an oval form, and a radiated white cicatrix in the centre, just about tha central body; the body being at the same time *immediately* under the peritoneal coat. This last is much insisted upon by Robert Lee, as he avers that "*false* corpora lutea are never observed in immediate connection with the peritoneum, a small portion of stroma intervening." As to the females, the first had been in a weakly state for some years, during which time she had *no* children. The second was unmarried, and had menstruated three days previous to her death. There was no history of the third case, but all the organs were healthy and the fallopian tube and uterus were in every way natural. Dr. Davidson expresses his confident opinion, that in none of these cases had there been impregnation previous to the appearance of these bodies. He refers to Professors Allison, Allen Thomson, John Reid, and Mr. Goodsir, in proof of the correctness of his statement, and of their perfect resemblance to a true corpus luteum.

Dr. Davidson, as the result of his investgations, says, "I am led to believe, *that impregnation cannot take place without the*

*appearance of a true corpus luteum, but that a true corpus luteum may appear independent of impregnation.*"—*Lond. & Edin. Month. Med. Journ.* Dec. 1841.

